# First person – Brian Belyea

**DOI:** 10.1242/dmm.038463

**Published:** 2018-12-18

## Abstract

First Person is a series of interviews with the first authors of a selection of papers published in Disease Models & Mechanisms, helping early-career researchers promote themselves alongside their papers. Brian Belyea is first author on ‘Leukemia development initiated by deletion of *RBP-J*: mouse strain, deletion efficiency and cell of origin’, published in DMM. Brian is Assistant Professor in the lab of Ariel Gomez at University of Virginia School of Medicine, Charlottesville, USA, investigating developmental disorders and disease states of the kidney and hematopoietic systems.


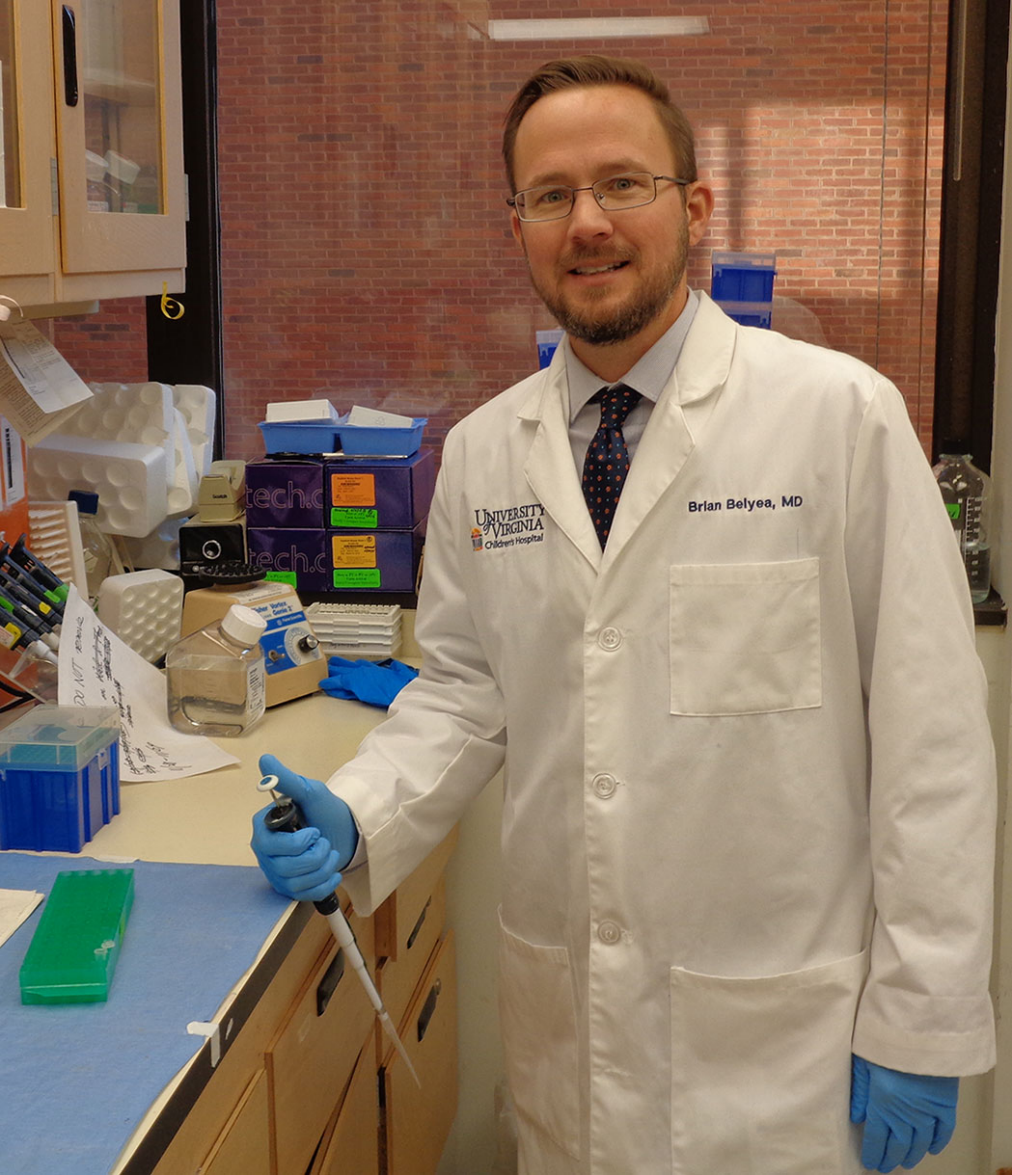


**Brian Belyea**

**How would you explain the main findings of your paper to non-scientific family and friends?**

B-cell leukemia is the most common malignancy of childhood. While cure rates have improved significantly over the last several decades, B-cell leukemia remains the leading cause of cancer-related deaths in children. Studies on the origin of B-cell leukemia in humans are challenging because patients are diagnosed at late stages of disease, prohibiting investigation into the initiating events. Our lab has developed a genetic mouse model of precursor B-cell leukemia, which recapitulates many features of childhood disease. Using this model, we are investigating the initiating events that drive B-cell development. In this work, we found that development of leukemia is influenced by several factors, including mouse strain, gene deletion efficiency and cell of origin.

**What are the potential implications of these results for your field of research?**

First, there are broad implications for scientists working with genetic mouse models, in that disease models must be carefully examined for confounding factors. In this work, we found distinct phenotypes with different mouse strains and different cells of origin. Second, there are more specific implications for scientists studying leukemia. We found that a unique subset of B-cell progenitors – renin-expressing cells – can be transformed into leukemia cells by deletion of the Notch signaling mediator *RBP-J*. However, deletion of *RBP-J* more broadly in B cells (using CD19 or Mb1 conditional deletion) does not lead to leukemia development. This suggests that renin progenitors are a particularly vulnerable cell type for neoplastic transformation and, further, not all cells of origin are created equal in their ability to become leukemia cells.

“An advantage of this genetic mouse model of B-cell leukemia is that we can investigate specific factors that might influence disease development.”

**What are the main advantages and drawbacks of the model system you have used as it relates to the disease you are investigating?**

An advantage of this genetic mouse model of B-cell leukemia is that we can investigate specific factors that might influence disease development. For example, in this study, we were able to perform conditional deletion of Notch in different B-cell subsets. Further, we were able to create more efficient deletion of Notch signaling by using two copies of Cre recombinase. Finally, the use of a fluorescence reporter mouse line allows us to track individual cells that have undergone Cre-mediated gene deletion. On the other hand, the use of a mouse model to study human disease does have limitations, including relevance. For example, we used our model to generate conditional deletion of Notch in two different strains of mice. Surprisingly, we found two different phenotypes. Thus, one must be careful with broad application of mouse findings to humans.

**What has surprised you the most while conducting your research?**

The biggest surprise from our findings is that renin-expressing B cells are uniquely vulnerable to leukemic transformation. In our initial studies, we found that conditional deletion of the Notch mediator *RBP-J* within a subset of B-cell progenitors that express renin leads to B-cell leukemia. We assumed that deletion of *RBP-J* within a broader pool of B cells, using CD19-Cre and Mb1-Cre, would similarly lead to development of B-cell leukemia. Surprisingly, none of these animals developed B-cell leukemia.

**Figure:**
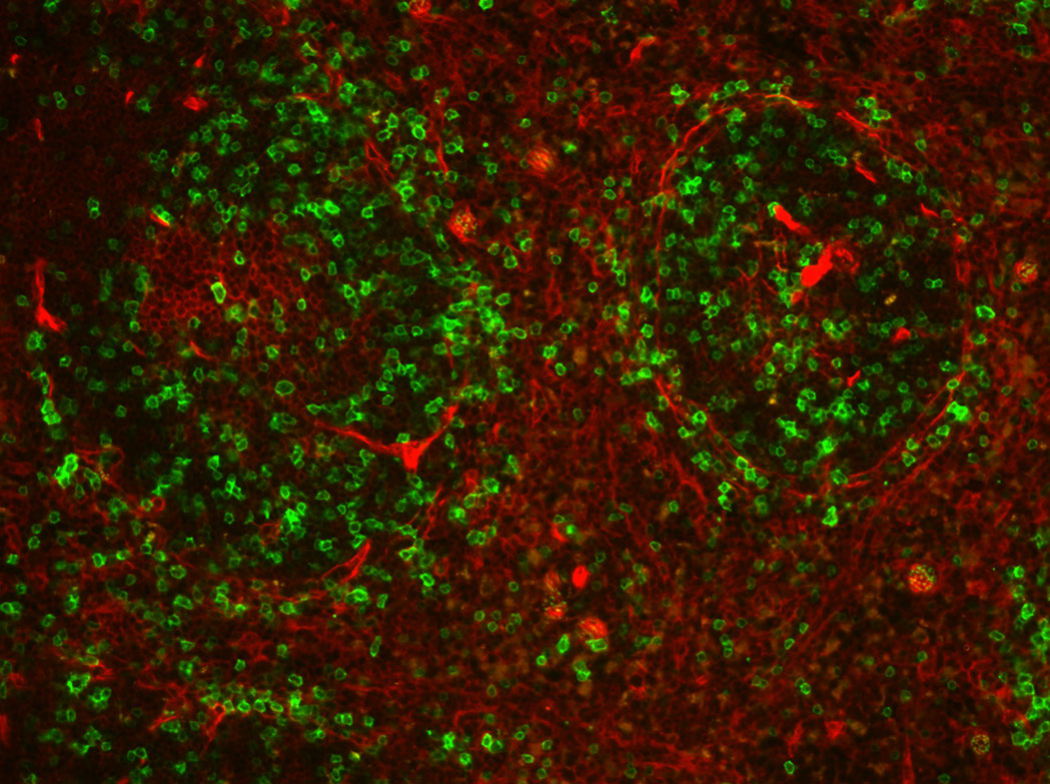
**GFP marks renin-lineage B cells in the spleen of a *Ren1^dCre/+^;mTmG* reporter mouse.** These unique progenitor cells are the cell of origin for precursor B-cell leukemia when the Notch effector gene *RBP-J* is deleted.

**Describe what you think is the most significant challenge impacting your research at this time and how will this be addressed over the next 10 years?**

A significant challenge that we face is translating these findings to humans. As mentioned above, the use of genetic mouse models provides opportunities to study the initiating events during leukemia genesis and follow individual cells over time as they become leukemia cells. However, studying these factors in human disease is challenging. I believe advances in molecular analysis, including single-cell mapping of gene expression and barcoding approaches, will allow one to detect the lineage history of an individual cancer cell to determine the precise cell of origin and the initiating genetic events.

**What changes do you think could improve the professional lives of early-career scientists?**

The answer here has to be increased opportunities for funding. With some funding rates as low as 10%, early-career scientists must apply for ten grants in order to get just one. This equals months and months of grant writing, which directly takes away from time in the lab learning techniques and performing experiments.

**What's next for you?**

One of the most intriguing findings of this paper is that renin-expressing cells are uniquely vulnerable to leukemic transformation following deletion of *RBP-J*. This result was surprising to us and suggests that not all progenitor cells are equal in terms of their ability to be transformed. We are excited to pursue these findings further, including investigating what is the exact identity of renin-expressing B cells and what is the function of these cells during normal hematopoiesis.
